# The Use of a Light-Emitting Diode Device for Neck Rejuvenation and Its Safety on Thyroid Glands

**DOI:** 10.3390/jcm10081774

**Published:** 2021-04-19

**Authors:** Young In Lee, Eunjung Lee, Kee-Hyun Nam, Dong Yeob Shin, Jihee Kim, Jangmi Suk, Jin Young Kwak, Ju Hee Lee

**Affiliations:** 1 Department of Dermatology, Severance Hospital, Cutaneous Biology Research Institute, College of Medicine, Yonsei University, Seoul 03722, Korea; ylee1124@yuhs.ac (Y.I.L.); mygirljihee@yuhs.ac (J.K.); 2 Scar Laser and Plastic Surgery Center, Yonsei Cancer Hospital, College of Medicine, Yonsei University, Seoul 03722, Korea; 3 Department of Computational Science and Engineering, Yonsei University, Seoul 03722, Korea; eunjunglee@yonsei.ac.kr; 4 Department of Surgery, Severance Hospital, Cutaneous Biology Research Institute, College of Medicine, Yonsei University, Seoul 03722, Korea; KHNAM@yuhs.ac; 5 Department of Internal Medicine, Severance Hospital, College of Medicine, Yonsei University, Seoul 03722, Korea; SHINDONGYI@yuhs.ac; 6 Global Medical Research Center, Seoul 06035, Korea; rose@gmrc.co.kr; 7 Department of Radiology, Severance Hospital, Research Institute of Radiological Science, Yonsei University College of Medicine, Seoul 03722, Korea

**Keywords:** LED device, low-level laser therapy, rejuvenation, safety, thyroid gland

## Abstract

Home-use light-emitting diodes (LEDs) are attracting growing attention regarding their anti-aging effects. Although most previous studies on the use of LED devices as a form of low-level laser therapy reported no significant adverse events, questions regarding the safety of using a light source on secretory tissues have been raised. This study aimed to assess the safety and efficacy of a home-use LED device for neck skin rejuvenation, particularly regarding its effect on thyroid gland morphology and function. Thyroid function tests and ultrasonographic analyses showed no significant changes after 16 weeks of LED use. Evaluation using the Lemperle wrinkle scale and global improvement scales by both investigators and subjects showed significant improvement after 16 weeks of daily application, as well as 8 weeks after discontinuation. Biophysical parameters, such as hydration, elasticity, and density, also showed significant improvements. Hence, the long-term use of the LED device was safe and effective for neck rejuvenation, and showed no significant side effects on the adjacent thyroid and parathyroid glands.

## 1. Introduction

A light-emitting diode (LED) emits a narrow spectrum of non-coherent light in the near-infrared, visible, and ultraviolet (UV) ranges [1]. Its penetration depth for inducing a therapeutic effect on tissues depends on its wavelength, which varies between 400 and 470 nm (blue light), 570 and 590 nm (yellow light), 630 and 700 nm (red light), and 800 and 1200 nm (near-infrared light) [2]. LEDs are used for low-level light therapy (LLLT) and photodynamic therapy for various skin conditions [1]. The introduction of photobiomodulation, which includes low-energy laser treatment or LED phototherapy, has shown positive effects in skin rejuvenation [3]. Previous studies observed increased adenosine triphosphate production, modulation of intracellular oxidative stress, induction of transcription factors, and increased blood flow after LED phototherapy [4]. Recently, Li et al. described the anti-inflammatory effect of a 630 nm LED via inhibition of reactive oxygen species and regulation of the nuclear factor-κB signaling pathway in human monocytes [5]. In addition, Park et al. demonstrated increased synthesis of type 1 procollagen and decreased expression of matrix metalloproteinases (MMPs) 1 and 2 in skin fibroblasts after irradiation with a 633 nm LED, suggesting possible mechanisms underlying the effects of LED irradiation in anti-aging and skin rejuvenation [6].

Among the available wavelengths, red LED has been shown to increase collagen type 1 and MMP-9 expression, which allow the degradation of fragmented collagen, leading to neocollagenesis. The near-infrared LED, on the other hand, has been shown to induce the release of guanylate cyclase and nitrous oxide, promoting vasodilation, growth factor production, and accelerated wound healing [5]. Recently, there has been a growing market demand for home-use cosmetic devices, and several clinical studies have successfully proven the effects of home-use LED mask devices for facial rejuvenation. With the development of the LED cosmetic industry, not only facial LED masks but also specialized forms of LED devices designed to improve body contour and neck folds have been manufactured.

Although red and near-infrared LEDs do not show harmful ionizing effects, questions have been raised regarding the use of phototherapy on secretory tissues as a low-intensity light source can trigger changes in their secretory patterns [7]. Concerns regarding the safety of internal organs, especially regarding the secretion patterns of the thyroid gland, have been raised specifically with respect to the use of LED neck rejuvenation devices. Höfling et al. assessed the effects of LLLT on thyroid vascularization in patients with autoimmune hypothyroidism and observed, via Doppler ultrasonography (US), improved thyroid parenchyma vascularization after LLLT [8]. A consequent placebo-controlled clinical study on the effect of LLLT in the treatment of hypothyroidism induced by chronic autoimmune thyroiditis suggested that photobiomodulation therapy was effective at improving thyroid function [9]. Serra et al. evaluated the possible changes in weight and secretory patterns of the thyroid and parathyroid glands irradiated with 780 nm LED in an animal model (Wistar rats) and found minimal and transient alterations in the hormone secretory patterns after irradiation, with normalization of all parameters after 2 months [7].

This study aimed to assess the safety of a 630 nm/850 nm home-use LED neck device on the function and morphology of the thyroid and parathyroid glands, and its efficacy in improving neck skin appearance and biophysical parameters. The neck device was applied daily for 16 weeks to observe its long-term safety and efficacy. After discontinuation of the device, participants were followed-up for 8 weeks to investigate any lasting effects after treatment.

## 2. Materials and Methods

### 2.1. Study Participants

Adult women with clinically visible skin aging or wrinkles on the neck were eligible for this study. Those with a mild-to-severe degree of neck wrinkles according to the investigators’ assessment of the neck fold severity rating (Lemperle wrinkle assessment scale ≥2) were enrolled. Participant exclusion criteria included a history of benign or malignant thyroid diseases (excluding asymptomatic cysts or nodules incidentally found in US), keloid scarring, previous inflammatory or infectious skin diseases, uncontrolled medical illness, pregnancy, and the use of any cosmetics containing growth factor-related constituents within six months before the treatment. This clinical trial was approved by the Institutional Review Board of Severance Hospital, Yonsei University, College of Medicine (IRB No. 4-2019-1174). The protocol was initiated after written informed consent was obtained from all participants, and the study was conducted in adherence to the principles of the Declaration of Helsinki.

### 2.2. Study Design

**Figure 1:**
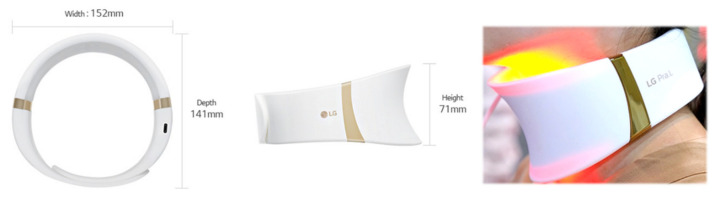
The home-use light-emitting diode (LED) neck device (SWL1, LG Electronics, Seoul, Korea).

The study was designed as a prospective single-arm interventional study. A total of 30 participants were instructed to wear the home-use neck LED device (SWL1, 630 nm/850 nm LED, LG Electronics, Seoul, Korea) daily for 9 min (Figure 1). The device was equipped with 120 LEDs, with wavelengths of 630 nm and 850 nm (60 LEDs for 630 nm and 60 LEDs for 850 nm). The total irradiance was 25 mW/cm^2^ (each wavelength emitting 50% of the total irradiance) with a maximum temperature of 38 °C. The net weight of the device was 220 g, and its size was 127 × 71 × 134 mm^3^ (width × height × diameter). All participants received daily treatment for 16 weeks before discontinuation of the treatment. After discontinuation, participants were followed-up for 8 weeks. The primary outcome of the study was the assessment of changes in thyroid and parathyroid function and their images under US. Laboratory blood tests for thyroid and parathyroid hormones and antibody levels, in addition to color US, were performed at baseline, 16 weeks after initiation of the treatment, and 8 weeks after the discontinuation of the treatment. A total of 90 real-time US examinations were performed by an experienced radiologist. Evaluation of neck biophysical parameters and clinical improvement was performed at baseline; after 4, 8, and 16 weeks of treatment; and 8 weeks after the discontinuation of treatment. 

### 2.3. Thyroid Function Analysis

Venous blood samples were collected from each subject after fasting for 8 h. The supernatant was centrifuged and stored in a low-temperature refrigerator at −80 °C until it was analyzed to measure thyroid function. Triiodothyronine (T3), thyroxine (T4), free T4, thyroid stimulating hormone (TSH), anti-thyroglobulin antibody (Ab), anti-thyroid peroxidase (anti-TPO) Ab, and parathyroid hormone (PTH) levels were measured. All blood test results were reviewed by an endocrinologist at Yonsei University who specialized in thyroid diseases.

### 2.4. Gray-Scale US and Power Doppler US

A majority of the real-time US examinations (*n* = 86) of the neck area were performed with a 5–12 MHz linear transducer (EPIQ5; Philips Healthcare, Bothell, WA, USA) by a radiologist with 22 years of experience in thyroid imaging. A few US examinations (*n* = 4) were performed with an identical transducer by a radiologist with 18 years of experience in thyroid imaging. The settings of the scanner were kept uniform throughout the study, including those of gain and time-gain compensation. After gray-scale US, power Doppler US was performed by the same radiologists. Power Doppler examinations were performed at standard settings for the thyroid glands, and the power Doppler amplification was set just under the level where the background noise disappeared. 

Power Doppler studies as well as gray-scale US studies were prospectively assessed and recorded. The thyroid parenchyma was analyzed for US features of diffuse thyroid disease when the thyroid gland showed one or more of the following suspicious findings: diffuse heterogeneous echogenicity or diffuse hypoechogenicity with/without micronodules, thyroid enlargement (anteroposterior diameter of the thyroid >2 cm on a longitudinal scan), multiple linear echogenicity, and scattered microcalcifications [10]. Thyroid parenchymal vascularity was assessed as either normal or increased, and thyroid nodules were categorized according to the 2015 American Thyroid Association Management Guideline [11]. We also evaluated parathyroid lesions and suspicious lymph nodes.

**Figure 2:**
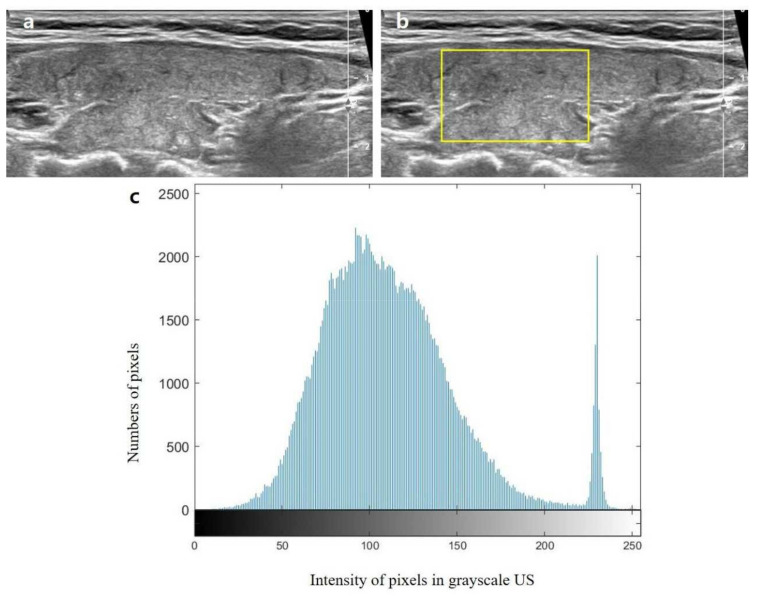
Example of texture analysis of gray-scale ultrasound (US) (on subject 18). (**a**) One longitudinal gray-scale US image was selected by an experienced radiologist. (**b**) The region of interest (ROI) was set to include a sufficient amount of thyroid parenchyma and was drawn by the radiologist. (**c**) From these ROIs, histogram parameters were automatically calculated with an in-house built software, which demonstrated the distribution of the number pixels (y-axis) according to the pixel intensity value (x-axis) within the ROIs.

To objectively evaluate and compare diffuse thyroid diseases, we recorded five histogram parameters, the mean, standard deviation, skewness, kurtosis, and entropy that were calculated as previously described [12]. The mean was defined as the average value of pixel intensity, whereas the standard deviation was defined as the deviation of pixel intensity. Skewness was defined as the distribution asymmetry about the mean, kurtosis was defined as the peakedness of the distribution, and entropy was defined as a measure of texture irregularity. Regions-of-interest for gray-scale US images were drawn to include more than 1/3 of a thyroid lobe so that a sufficient amount of the underlying thyroid parenchyma was included while excluding thyroid nodules. An example of texture analysis on gray-scale US is shown on Figure 2. 

### 2.5. Neck Rejuvenation and Wrinkle Assessment

Efficacy of neck rejuvenation was assessed at baseline; after 4, 8, and 16 weeks of treatment; and at 8 weeks after the discontinuation of treatment. The relative skin hydration status and skin barrier function were measured using a Corneometer (Courage Khazaka Electronics, Köln, Germany). Changes in skin elasticity after LED treatment were measured using the Cutometer Dual MPA580 (Courage Khazaka Electronics, Köln, Germany) and Torsional Ballistometer BLS 750 (Dia-Stron Limited, Hampshire, UK). Ultrascan was adopted to observe changes in skin density (Courage + Khazaka Electronic GmbH, Köln, Germany), and three-dimensional (3D) scanning using Morpheus 3D Scanner^®^ (Morpheus Co., Ltd., Seoul, Korea) and Antera 3D™ (Miravex, Dublin, Ireland) was used for 3D contour visualization of the neck. Each measurement was repeated three times at every visit.

Standardized photographs were uniformly taken without changes in camera settings at each visit using a Canon 800D DSLR camera (Canon Inc., Tokyo, Japan). Clinical outcomes were assessed by three independent, blinded dermatologists according to the Lemperle wrinkle assessment scale by comparing the standardized clinical photographs of the neck as previously described [13] (0 = no wrinkles, 1 = barely perceptible wrinkle, 2 = shallow wrinkle, 3 = moderately deep wrinkle, 4 = deep wrinkle, well-defined edges, 5 = very deep wrinkle, redundant fold). In addition, the overall improvements in wrinkles were also assessed by both blinded and independent dermatologists and by the participants, using the global improvement scale (GIS: grade 1 = worse; grade 2 = no change; grade 3 = somewhat improved; grade 4 = moderately improved; grade 5 = very much improved).

### 2.6. Statistical Analyses

Statistical analyses of data from the clinical study were performed with SPSS v. 25.0 (SPSS Inc., Chicago, IL, USA). For quantitative variables, a repeated-measure analysis of variance (RM-ANOVA) followed by post hoc tests via Bonferroni correction was carried out among each visit (data shown as mean ± SEM). For categorical non-parametric variables, including Lemperle wrinkle assessment scale, Wilcoxon signed rank test using Bonferroni’s correction was performed to account for multiple comparisons. A *p*-value < 0.05 was considered statistically significant. 

## 3. Results

### 3.1. Study Participants

**Figure 3:**
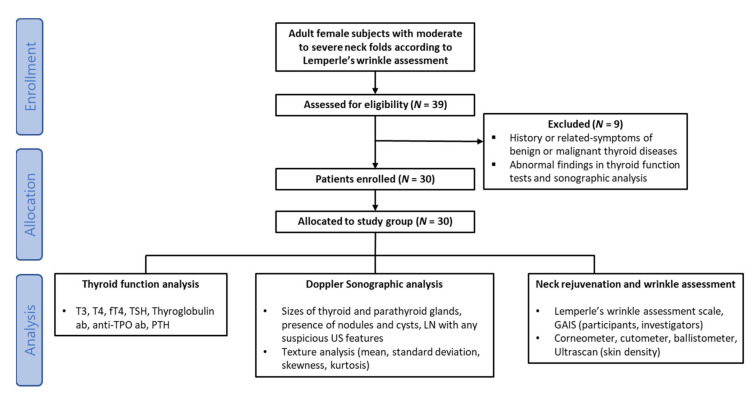
Flow-chart of participant screening and study design. T3, triiodothyronine; T4, thyroxine; fT4, free thyroxine; TSH, thyroid stimulating hormone; anti-TPO, anti-thyroid peroxidase antibody; PTH, parathyroid hormone.

Out of the 39 patients who were screened, 30 adult women with clinically visible skin aging or wrinkles on the neck were deemed eligible for this study; those between the ages of 30–47 (mean 43.9) years with Fitzpatrick phototypes III to V were enrolled (Figure 3). The initial neck fold severity of the study population, evaluated according to the Lemperle wrinkle assessment scale, was 2.66 ± 0.66. Among the nine women who were excluded from the study during the screening process, two were excluded due to incidental findings of suspicious nodules in the thyroid gland. One woman agreed to undergo fine-needle aspiration biopsy and was diagnosed with a carcinoma; the other refused to undergo biopsy. Seven more women were excluded due to abnormal findings on blood tests: five showed abnormally elevated thyroglobulin Ab levels, and two had abnormally elevated TSH levels. No participants dropped out from the study after enrollment.

### 3.2. Assessment of Changes in Thyroid Function

**Figure 4:**
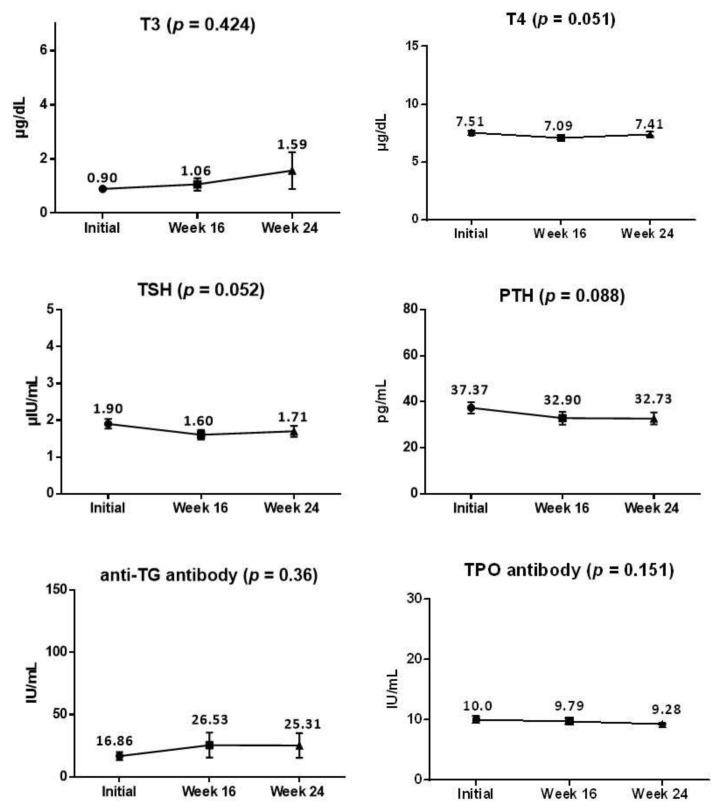
Changes in thyroid function tests during the study (data are shown as mean ± SEM). T3, triiodothyronine; T4, thyroxine; TSH, thyroid stimulating hormone; PTH, parathyroid hormone; anti-TG, anti-thyroglobulin; anti-TPO, anti-thyroid peroxidase.

The normal ranges of the thyroid function tests provided from our institution were as follows: T4, 5.26–9.77 μg/dL; T3, 0.61–1.70 ng/mL; free T4, 0.80–1.23 ng/dL; TSH, 0.41–4.30 μIU/mL; thyroglobulin Ab, 0–130.6 IU/mL; anti-TPO Ab, 0–13.7 IU/mL; and PTH, 15–65 pg/mL. Among the participants enrolled, eight showed slightly elevated initial anti-TPO Ab or PTH levels (*n* = 3), or lower initial free T4 or T4 levels (*n* = 5), without related symptoms or past medical histories. These values were thoroughly reviewed by the experienced endocrinologist and were categorized as “not clinically significant.” During the 16 weeks of using the study device and the 8-week follow-up period after discontinuation, the mean thyroid and PTH levels, as well as the Ab levels (thyroglobulin Ab, anti-TPO Ab), did not change significantly (Figure 4, RM-ANOVA).

### 3.3. Assessment of the Changes in Thyroid US Findings

The US findings of the thyroid and parathyroid glands showed no significant changes in volumes, echogenicity, lymph nodes (LNs), blood flow, or number and size of incidentalomas. The initial findings of four enrolled participants revealed heterogeneous echogenicity of the glands, and 13 participants showed incidental findings of asymptomatic benign nodules. Those with heterogenous echogenicity did not develop thyroid-related symptoms and the laboratory blood tests remained within the normal ranges during the 6-month study period. The texture analysis on thyroid glands also showed no significant changes in mean, standard deviation, skewness, or kurtosis during the study period (Table 1, RM-ANOVA).

As for incidentalomas in the enrolled participants, the initial size of the biggest nodule was 24 mm, and the smallest size was 3 mm. The assessment of the US findings during the entire study period revealed no significant changes in the size and number of incidental thyroid nodules. Notably, one of 13 participants who had asymptomatic nodules showed increased PTH levels during the use of the study device, from 43.4 pg/mL on the initial visit to 81.7 pg/mL after 16 weeks, and 73.6 pg/mL after 8 weeks of discontinuation. Nonetheless, the participant did not have abnormal blood flow or echogenicity of the thyroid gland, and all other laboratory tests results were within the normal ranges. Additional laboratory testing on the participant showed low 25-OH-Vitamin D level (10.33 ng/mL), suggesting an incidental abnormal PTH finding due to vitamin D insufficiency.

### 3.4. Assessment of Neck Rejuvenation and Wrinkle Reduction

**Figure 5:**
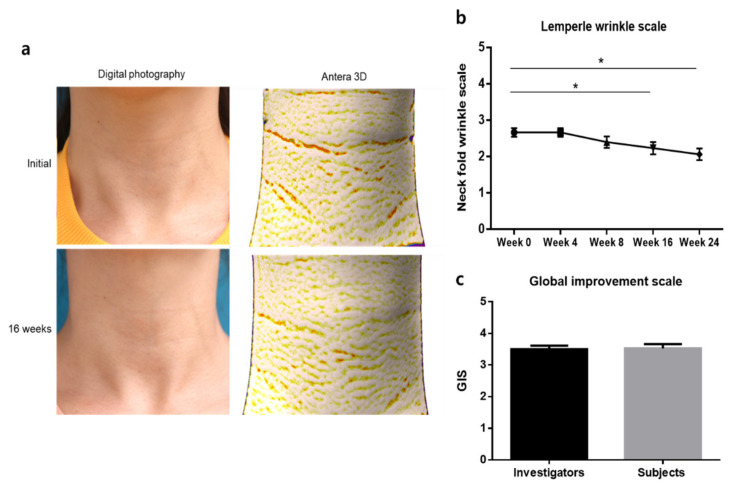
Improvement in neck wrinkles during the study. (**a**) Comparison of clinical photographs and Antera 3D images at baseline and at week 16. (**b**) Changes in Lemperle scale scores during the study period. (**c**) Global improvement scale scores assessed by investigators and participants. * *p* < 0.05, Wilcoxon singed rank test.

Neck rejuvenation and wrinkle reduction were evaluated by three independent dermatologists by blindly reviewing the clinical photographs and Antera 3D™ images up to the time of treatment. The investigator’s assessment scale score of horizontal neck folds (Lemperle scale) was significantly reduced after 16 weeks of using the study device (Figure 5a,b, Wilcoxon signed rank test, *p* < 0.001). After 8 weeks of discontinuation of the device, the Lemperle scale score remained significantly lower than the initial scale of the neck folds (Figure 5b, Wilcoxon signed rank test, *p* < 0.001). The GIS evaluated by the investigators was 3.50 ± 0.63, whereas that evaluated by the participants was 3.53 ± 0.13, both indicating “mild improvement” (Figure 5c).

**Figure 6:**
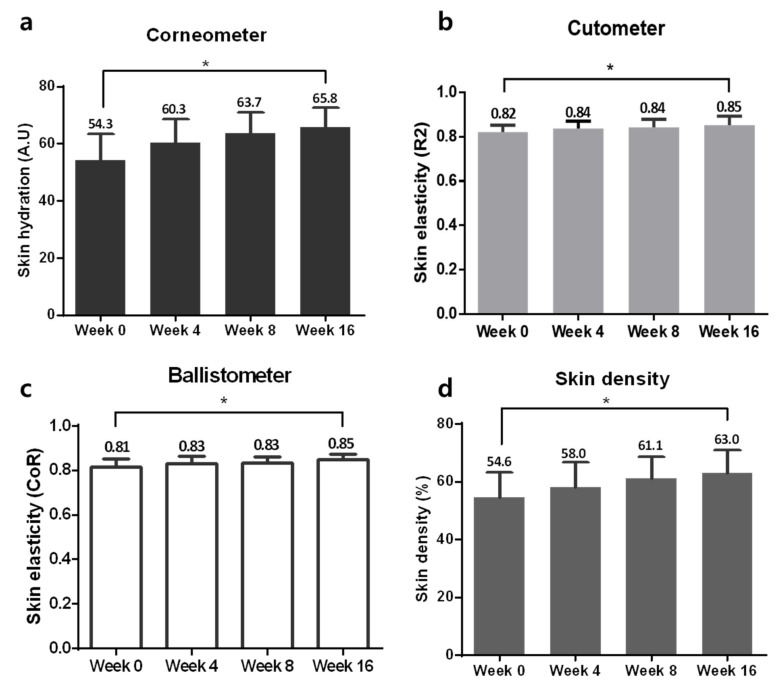
Improvements in neck rejuvenation. (**a**) Changes in skin hydration on a Corneometer. (**b**) Changes in skin elasticity on a Cutometer. (**c**) Changes in skin elasticity on a Ballistometer. (**d**) Changes in skin density on Ultrascan during the study period. * *p* < 0.05, repeated-measure analysis of variance (RM-ANOVA).

Changes in skin hydration were measured with a Corneometer and showed significant improvement during the 16 weeks of using the study device (Figure 6a, RM-ANOVA, *p* < 0.01). Skin elasticity was evaluated by the R2 and CoR values from a Cutometer and a Ballistometer, and both values were increased after 16 weeks (Figure 6b,c, RM-ANOVA, *p* < 0.01). Skin density was measured by Ultrascan and was significantly increased after 16 weeks of device application (Figure 6d, RM-ANOVA, *p* < 0.01).

## 4. Discussion

The use of LED home-use devices has gained significant interest over the past few years in the anti-aging cosmeceutical market. These LLLT devices are sources of photobiomodulation therapy that have been used not only to promote wound healing, but also to produce anti-inflammatory effects, photo-rejuvenation, as well as for the treatment of various dermatological disorders [14]. In particular, red and near-infrared LEDs can be used as skin biostimulators to decelerate fibroblast aging by exerting antioxidative and collagen-enhancing activities [15,16]. Recently, the easy accessibility of LED devices by consumers and their relatively low cost compared to those of frequent visits to a dermatologic clinic have stimulated the development of numerous LED-based home devices for rejuvenation and anti-aging action.

Although the efficacy and safety of many home-use LED devices have been examined in a wide range of applications, including anti-aging and reduction in facial wrinkles, most studies reported positive results without any adverse events [5,17]. Nevertheless, a recent case study reported retinal damage due to prolonged exposure to a blue light home-use LED face mask [17]. Our study, to the best of our knowledge, is the first prospective clinical report on the safety of internal organs, such as the thyroid and parathyroid glands, after long-term use of home-use red to near-infrared LED devices for skin rejuvenation. Compared to the average skin thickness of the cheek and lower eyelid reported previously (respectively, 3.22 ± 0.63 and 2.19 ± 0.48) the reported average skin thickness of the lateral neck is 1.50 ± 0.82 [18]. Therefore, it is easier for red to near-infrared LED light to penetrate the relatively thin skin on the neck and affect the adjacent internal organs, such as the thyroid gland.

After 6 months of follow-up, our study showed that 16 weeks of daily use of the LED neck device did not alter the function and sonographic findings of the thyroid and parathyroid glands. The additional 8-week follow-up after discontinuation of the use of the study device also revealed no significant differences in thyroid hormones, PTH levels, Ab levels, vascularity, size of the glands, LNs, incidentalomas, or texture analysis. One of the participants had an incidental finding of low 25-hydroxy vitamin D level during the study, after the observation of elevated PTH levels during the use of the study device. After close review of the patient’s medical records by an experienced endocrinologist, the elevated PTH level was determined to be due to an underlying vitamin D insufficiency, rather than to the use of the LED neck device.

Although the corresponding data are not shown in this study, the measurement of collagen synthesis in normal human dermal fibroblasts via Procollagen type I C-peptide ELISA kit showed increased collagen production after exposing 2.0 × 10^4^ cells to 25 mW/cm^2^ of red to near-infrared LED from a distance of 8 cm from the culture plates. A subsequent animal study on mini pigs also showed increased skin density after 8 weeks of irradiation, 3 times a week, using an LED of the same specification as the study device (manuscript in preparation). In this study, the daily use of the LED neck device for 16 weeks resulted in significantly increased skin density, hydration, and elasticity compared with those in the baseline. Both the investigators and participants observed a “mild improvement” in neck wrinkles via GIS after using the device. The measurements after discontinuation of the study device for 8 weeks also showed significantly improved skin hydration, elasticity, and density, indicating a long-term effect of the daily use of this home-use LED device on skin rejuvenation.

This study is the first long-term observation on the safety and efficacy of a home-use LED neck rejuvenation device. It specifically focused on the safety on the thyroid and parathyroid glands by evaluating possible effects of the red to near-infrared LED on their functions, vascularity, adjacent LNs, and benign nodules and cysts. As the study’s limitations, along with a lack of the control group, we note that although the investigators exclusively enrolled healthy adult women without a previous thyroid medical history or associated symptoms, nine of the 39 women (23%) failed screening due to abnormal blood tests or sonographic findings. Previous studies confirm the relatively high incidence of thyroid incidentalomas; a large Korean study showed a prevalence of thyroid nodules or cysts of 34% among subjects undergoing thyroid ultrasonography during a routine health exam [19]. A prevalence of incidentalomas as high as 67% on ultrasonography has also been reported [20]. Hence, although home-use LED devices are advertised to be used by healthy consumers, the fact that those with underlying thyroid diseases can also be exposed to these devices should not be neglected. Further studies on the safety of LED devices for subjects with known thyroid disease are needed to explore the safety of home-use LEDs in the general population.

In conclusion, our long-term study demonstrated the efficacy of a home-use LED device for neck skin rejuvenation and its safety. This study, for the first time, investigated the possible effects of the anti-wrinkle LLLT device on the adjacent secretory organs, thyroid and parathyroid glands, and revealed no significant side effects.

## Figures and Tables

**Table 1:** Changes in sonographic findings during the study.

Parameters	Mean ± SD	*p*-Value
Thyroid volume (mL)		*p* = 0.371
Initial	8.60 ± 3.03	
4 months	8.55 ± 3.02	
6 months (discontinued for 2 months)	8.44 ± 2.89	
Underlying echogenicity (N)		N/A
Normal	26	
Heterogeneous	4	
Mean		*p* = 0.732
Initial	107.80 ± 14.43	
4 months	108.56 ± 15.70	
6 months (discontinued for 2 months)	110.60 ± 15.84	
Standard deviation		*p* = 0.630
Initial	31.45 ± 3.60	
4 months	31.93 ± 3.70	
6 months (discontinued for 2 months)	31.04 ± 3.79	
Skewness		*p* = 0.231
Initial	0.25 ± 0.20	
4 months	0.32 ± 0.19	
6 months (discontinued for 2 months)	0.25 ± 0.22	
Kurtosis		*p* = 0.687
Initial	3306.80 ± 580.25	
4 months	3368.24 ± 446.53	
6 months (discontinued for 2 months)	3251.87 ± 578.83	
Parathyroid (N)		N/A
Normal	30	
Abnormal	0	
Lymph nodes (N)		N/A
Normal	30	
Abnormal	0	
Nodules (N)		N/A
None	17	
Single nodule	6	
Multiple nodules	7	
